# High-Pressure Synthesis and Crystal Structure of Ce_4_B_14_O_27_

**DOI:** 10.1002/zaac.201200402

**Published:** 2012-10-24

**Authors:** Ernst Hinteregger, Lukas Perfler, Hubert Huppertz

**Affiliations:** [a] Institut für Allgemeine, Anorganische und Theoretische Chemie, Leopold-Franzens-Universität InnsbruckInnrain 80-82, 6020 Innsbruck, Austria; [b] Institut für Mineralogie und Petrographie, Leopold-Franzens-Universität InnsbruckInnrain 52, 6020 Innsbruck, Austria

**Keywords:** High-pressure chemistry, Multianvil, Cerium, Borates, Crystal structure

## Abstract

Ce_4_B_14_O_27_ was synthesized under conditions of 2.6 GPa and 750 °C in a Walker-type multianvil apparatus. The crystal structure was determined on the basis of single-crystal X-ray diffraction data, collected at room temperature, revealing that Ce_4_B_14_O_27_ is isotypic to La_4_B_14_O_27_. Ce_4_B_14_O_27_ crystallizes monoclinically with four formula units in the space group *C*2/*c* (No. 15) and the lattice parameters *a* = 1117.8(2), *b* = 640.9(2), *c* = 2531.7(5) pm, and *β* = 100.2(1)°. The three-dimensional boron-oxygen framework consists of [BO_4_]^5–^ tetrahedra and trigonal-planar [BO_3_]^3–^ groups. The structure contains two crystallographically different cerium ions. Furthermore, Raman spectroscopy was performed on single crystals of Ce_4_B_14_O_27_.

## Introduction

The structural chemistry of oxoborates exhibits a respectable diversity, which yields from the ability of the boron atom to form trigonal-planar [BO_3_]^3–^ groups and tetrahedral [BO_4_]^5–^ groups. These groups can occur isolated or linked to highly-condensed three-dimensional networks. In the majority of cases, the trigonal-planar [BO_3_]^3–^ groups disappear with increasing pressure, so in high-pressure oxoborates, the boron atoms favor the fourfold coordination forming [BO_4_]^5–^ groups. Above a pressure of 10 GPa, only a few compounds are known, which contain trigonal-planar [BO_3_]^3–^ groups, e.g. Ho_31_O_27_(BO_3_)_3_(BO_4_)_6_.^1^ The linking of the tetrahedral [BO_4_]^5–^ groups follows normally via common corners. In the past, we observed that these boron–oxygen tetrahedra can share common edges to realize denser structures like the polyborates *RE*_4_B_6_O_15_ (*RE* = Dy, Ho),^2^,^3^ and *α*-*RE*_2_B_4_O_9_ (*RE* = Sm–Ho).^4^–^6^ Moreover, high-pressure/high-temperature syntheses led to increased coordination numbers (CN) of the rare-earth ions, and also the coordination numbers of the oxygen atoms could be partially enhanced from twofold (O^2^) to threefold coordinated (O^3^).

Recent studies into the chemistry of rare-earth oxoborates under high-pressure/high-temperature conditions reached to a large number of polymorphs and new compositions. Before we started research, the system Ce_2_O_3_/B_2_O_3_ was represented by four modifications of the *ortho*-oxoborate CeBO_3_ (Ce_2_O_3_:B_2_O_3_ = 1:1; λ-, ν-, π-, and H-CeBO_3_),^7^–^11^ the *meta*-oxoborate *α*-Ce(BO_2_)_3_ (Ce_2_O_3_:B_2_O_3_ = 1:3),^1^,^12^ and β-*RE*B_5_O_9_.^13^ The application of high-pressure/high-temperature techniques allowed the synthesis of δ-Ce(BO_2_)_3_^14^ and *γ*-Ce(BO_2_)_3_,^15^ two new modifications of cerium *meta*-oxoborate. While the monoclinic δ-Ce(BO_2_)_3_ was synthesized at 3.5 GPa and 1050 °C, the synthesis of the orthorhombic γ-Ce(BO_2_)_3_ needed high-pressure/high-temperature conditions of 7.5 GPa and 1000 °C. Despite intensive search, no cerium-polyoxoborates with compositions like *RE*_4_B_10_O_21_ (*RE* = La, Pr),^16^,^17^ *RE*_3_B_5_O_12_ (*RE* = Er–Lu),^18^ or *RE*_4_B_6_O_15_ (*RE* = Dy, Ho)^2^,^3^ could be synthesized. Now, the application of high-pressure/high-temperature conditions enabled the synthesis of a cerium-polyoxoborate with the composition Ce_4_B_14_O_27_, which is isotypic to the recently discovered La_4_B_14_O_27_.^19^ In this paper, we describe the synthesis of Ce_4_B_14_O_27_, the single-crystal structure determination, Raman spectroscopic investigations, and a comparison to the isotypic phase La_4_B_14_O_27_.

## Experimental Section

**Synthesis:** During our attempts, to synthesize a cerium fluorido- or fluoride borate under high-pressure/high-temperature conditions of 2.6 GPa and 750 °C, the new cerium oxoborate Ce_4_B_14_O_27_ was synthesized, starting from a mixture of 79.2 mg CeO_2_ (Auer-Remy, Hamburg, Germany, 99.9 %), 80.1 mg B_2_O_3_ (Strem Chemicals, Newburyport, USA, 99.9+ %), and 90.7 mg CeF_3_ (Strem Chemicals, Newburyport, USA 99.9+ %). The starting materials were finely ground and filled into a boron nitride crucible (Henze BNP GmbH, HeBoSint® S100, Kempten, Germany). The crucible was placed into an 18/11-assembly and compressed by eight tungsten carbide cubes (TSM-10, Ceratizit, Reutte, Austria). To apply the pressure, a 1000 t multianvil press with a Walker-type module (both devices from the company Voggenreiter, Mainleus, Germany) was used. The assembly and its preparation are described in the literature.^20^–^24^

The 18/11 assembly was compressed up to 2.6 GPa in 65 min and heated to 750 °C (cylindrical graphite furnace) in the following 10 min, kept there for 15 min, and cooled down to 450 °C in 25 min at constant pressure. After natural cooling down to room temperature by switching off the heating, a decompression period of 3.5 h was required. The recovered octahedral pressure medium (MgO, Ceramic Substrates & Components Ltd., Newport, Isle of Wight, UK) was broken apart and the sample was carefully separated from the surrounding graphite and boron nitride. The compound Ce_4_B_14_O_27_ was found in the form of colorless air-resistant crystals.

Two corresponding experiments under ambient pressure conditions at 700 °C and 850 °C using CeO_2_, B_2_O_3_, and CeF_3_ (flux material) in a boron-nitride crucible did not lead to the desired polyborate Ce_4_B_14_O_27_. Instead of, the syntheses led to the monoclinic *meta*-borate α-Ce(BO_2_)_3_.^1^,^12^

**Figure 1:**
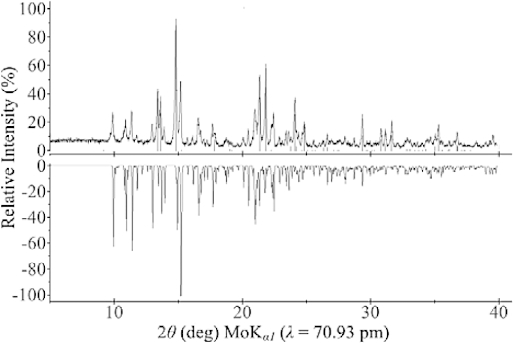
Experimental powder pattern of Ce_4_B_14_O_27_ (top) and the reflections of CeF_3_ (top, lines) in comparison with the theoretical powder pattern of Ce_4_B_14_O_27_ (bottom) based on single-crystal diffraction data.

**Crystal Structure Analysis:** The sample was characterized by powder X-ray diffraction, which was performed in transmission geometry on a flat sample of the reaction product, using a STOE STADI P powder diffractometer with Ge(111)-monochromatized Mo-*K_α_*_1_ (*λ* = 70.93 pm) radiation. The diffraction pattern showed reflections of Ce_4_B_14_O_27_ and CeF_3_. Figure 1 shows the experimental powder pattern that matches well with the theoretical pattern simulated from the single-crystal data. Small single-crystals of Ce_4_B_14_O_27_ were isolated by mechanical fragmentation. The single crystal intensity data were collected at room temperature with a Nonius Kappa-CCD diffractometer with graphite-monochromatized Mo-*K_α_* radiation (*λ* = 71.073 pm). A semiempirical absorption correction based on equivalent and redundant intensities (SCALEPACK)^[25]^ was applied to the intensity data. All relevant details of the data collection and evaluation are listed in Table 1. According to the systematic extinctions, the monoclinic space group *C*2/*c* (no. 15) was derived. Because of the fact that Ce_4_B_14_O_27_ is isotypic to La_4_B_14_O_27_,^16^ the structural refinement was performed using the positional parameters of La_4_B_14_O_27_ as starting values [SHELXL-97^26^–^28^ (full-matrix least-squares on *F*^2^)]. In comparison with the structure data of *Schleid* et al., in which only the lanthanum atoms could be refined with anisotropic displacement parameters, an anisotropic refinement for all atoms of Ce_4_B_14_O_27_ was possible. The final difference Fourier syntheses did not reveal any significant peaks in the refinement. As positional parameters of La_4_B_14_O_27_, we used the standard setting as deposited at the FIZ Karlsruhe with the deposition number CSD-418109 http://www.ccdc.cam.ac.uk/data_request/cif. Table 2, Table 3, Table 4, and Table 5 list the positional parameters, anisotropic displacement parameters, interatomic distances, and angles.

**Table 1:** Crystal data and structure refinement of Ce_4_B_14_O_27_

Empirical Formula	Ce_4_B_14_O_27_
Molar mass /g**·**mol^–1^	1143.82
Crystal system	monoclinic
Space group	*C*2/*c* (No. 15)
Single-crystal diffractometer	Bruker AXS / Nonius Kappa CCD
Radiation	Mo-*K_α_* (*λ* = 71.073 pm)
*a* /pm	1117.8(2)
*b* /pm	640.9(2)
*c* /pm	2531.7(5)
*β* /°	100.2(1)
Volume /Å^3^	1785.0(6)
Formula units per cell	4
Temperature /K	293(2)
Calculated density /g**·**cm^–3^	4.26
Crystal size /mm^3^	0.04 × 0.03 × 0.02
Absorption coefficient /mm^–1^	10.2
*F*(000)	2072
*θ* range /°	1.6–37.8
Range in *h k l*	–17 < *h* < 19
	–11 < *k* < 8
	–41 < *l* < 43
Total no. reflections	13907
Independent reflections	4768 (*R*_int_ = 0.0509)
Reflections with *I* > 2*σ*(*I*)	3605 (*R*_σ_ = 0.0598)
Data / parameters	4768 / 205
Absorption correction	Multi-scan^23^
Goodness-of-fit (*F*^2^)	1.058
Final *R* indices [*I* > 2*σ*(*I*)]	*R*_1_ = 0.0360; *wR*_2_ = 0.0600
*R* indices (all data)	*R*_1_ = 0.0613; *wR*_2_ = 0.0660
Largest differ. peak / deepest hole /e**·**Å^–3^	2.15 / –2.73

**Table 2:** Atomic coordinates and isotropic equivalent displacement parameters (*U*_eq_ /Å^2^) for Ce_4_B_14_O_27_ and (La_4_B_14_O_27_). *U*_eq_ is defined as one-third of the trace of the orthogonalized *U*_ij_ tensor

Atom	Wyckoff-Symbol	*x*	*y*	*z*	*U*_eq_
Ce1	8*f*	0.17020(2)	0.08703(3)	0.184732(6)	0.00727(5)
(La1)		(0.1709)	(0.0835)	(0.1849)	(0.0057)
Ce2	8*f*	0.09943(2)	0.24975(3)	0.443926(7)	0.00805(5)
(La2)		(0.0985)	(0.2487)	(0.4441)	(0.0067)
B1	8*f*	0.0925(3)	0.2682(5)	0.2948(2)	0.0067(6)
(B1)		(0.0899)	(0.2667)	(0.2948)	(0.0072)
B2	8*f*	0.2822(3)	0.0764(6)	0.3244(2)	0.0071(6)
(B2)		(0.2807)	(0.0759)	(0.3245)	(0.0059)
B3	8*f*	0.4010(3)	0.4345(6)	0.1494(2)	0.0074(6)
(B3)		(0.4005)	(0.4340)	(0.1493)	(0.0053)
B4	8*f*	0.3948(3)	0.2377(6)	0.0549(2)	0.0078(6)
(B4)		(0.3951)	(0.2385)	(0.0545)	(0.0047)
B5	8*f*	0.3778(3)	0.1689(6)	0.4217(2)	0.0088(6)
(B5)		(0.3788)	(0.1682)	(0.4213)	(0.0082)
B6	8*f*	0.1678(3)	0.1430(6)	0.0469(2)	0.0075(6)
(B6)		(0.1686)	(0.1428)	(0.0461)	(0.0084)
B7	8*f*	0.5038(3)	0.1001(6)	0.1868(2)	0.0075(6)
(B7)		(0.5053)	(0.1010)	(0.1873)	(0.0051)
O1	4*e*	0	0.2029(5)	¼	0.0106(6)
(O1)		(0)	(0.2025)	(¼)	(0.0093)
O2	8*f*	0.0634(2)	0.1574(4)	0.34220(8)	0.0070(4)
(O2)		(0.0629)	(0.1544)	(0.3417)	(0.0053)
O3	8*f*	0.0991(2)	0.4951(4)	0.30690(9)	0.0078(4)
(O3)		(0.0985)	(0.4916)	(0.3067)	(0.0083)
O4	8*f*	0.2149(2)	0.2163(4)	0.28251(8)	0.0080(4)
(O4)		(0.2139)	(0.2143)	(0.2831)	(0.0054)
O5	8*f*	0.2909(2)	0.3928(3)	0.16986(9)	0.0084(4)
(O5)		(0.2902)	(0.3919)	(0.1699)	(0.0080)
O6	8*f*	0.1091(2)	0.4888(3)	0.19066(9)	0.0073(4)
(O6)		(0.1102)	(0.4881)	(0.1912)	(0.0070)
O7	8*f*	0.3071(2)	0.2091(4)	0.37269(8)	0.0094(4)
(O7)		(0.3071)	(0.2077)	(0.3725)	(0.0074)
O8	8*f*	0.5040(2)	0.3111(4)	0.17882(9)	0.0082(4)
(O8)		(0.5033)	(0.3083)	(0.1787)	(0.0066)
O9	8*f*	0.3863(2)	0.4085(4)	0.09132(8)	0.0089(4)
(O9)		(0.3865)	(0.4067)	(0.0919)	(0.0069)
O10	8*f*	0.5006(2)	0.1005(4)	0.07423(9)	0.0089(4)
(O10)		(0.5009)	(0.1023)	(0.0737)	(0.0076)
O11	8*f*	0.2906(2)	0.0967(4)	0.04966(9)	0.0101(4)
(O11)		(0.2907)	(0.0994)	(0.0489)	(0.0082)
O12	8*f*	0.4093(2)	0.3303(4)	0.00230(9)	0.0117(4)
(O12)		(0.4092)	(0.3334)	(0.0026)	(0.0080)
O13	8*f*	0.1151(2)	0.1508(4)	0.09082(9)	0.0114(4)
(O13)		(0.1169)	(0.1496)	(0.0902)	(0.0098)
O14	8*f*	0.3279(2)	0.2033(4)	0.46524(9)	0.0125(5)
(O14)		(0.3279)	(0.2055)	(0.4644)	(0.0084)

**Table 3:** Anisotropic displacement parameters (*U*_ij_ /Å^2^) for Ce_4_B_14_O_27_ (space group *C*2/*c*)

Atom	*U*_11_	*U*_22_	*U*_33_
Ce1	0.00685(8)	0.00764(8)	0.00715(7)
Ce2	0.00801(8)	0.00791(8)	0.00758(8)
B1	0.005(2)	0.007(2)	0.009(2)
B2	0.006(2)	0.008(2)	0.007(2)
B3	0.008(2)	0.006(2)	0.008(2)
B4	0.008(2)	0.010(2)	0.005(2)
B5	0.011(2)	0.007(2)	0.008(2)
B6	0.009(2)	0.006(2)	0.008(2)
B7	0.009(2)	0.008(2)	0.006(2)
O1	0.013(2)	0.009(2)	0.009(2)
O2	0.008(2)	0.006(2)	0.0074(9)
O3	0.007(2)	0.0048(9)	0.011(2)
O4	0.008(2)	0.011(2)	0.0054(9)
O5	0.007(2)	0.006(2)	0.014(2)
O6	0.0043(9)	0.0065(9)	0.012(2)
O7	0.009(2)	0.012(2)	0.0068(9)
O8	0.007(2)	0.0058(9)	0.011(2)
O9	0.011(2)	0.008(2)	0.0074(9)
O10	0.0051(9)	0.010(2)	0.011(2)
O11	0.007(2)	0.008(2)	0.015(2)
O12	0.007(2)	0.020(2)	0.007(2)
O13	0.010(2)	0.017(2)	0.008(2)
O14	0.011(2)	0.019(2)	0.008(2)

	*U*_23_	*U*_13_	*U*_12_
Ce1	0.00049(6)	0.00082(5)	–0.00081(6)
Ce2	–0.00119(6)	–0.00043(6)	0.00000(6)
B1	0.001(2)	0.001(2)	0.000(2)
B2	–0.001(2)	0.001(2)	0.002(2)
B3	–0.001(2)	0.002(2)	–0.001(2)
B4	–0.001(2)	–0.001(2)	–0.001(2)
B5	–0.000(2)	0.001(2)	0.002(2)
B6	0.002(2)	0.003(2)	0.000(2)
B7	0.001(2)	0.001(2)	0.000(2)
O1	0	0.000(2)	0
O2	0.0003(8)	0.0035(8)	0.0013(8)
O3	–0.0004(8)	0.0015(8)	–0.0002(8)
O4	0.0005(8)	0.0021(8)	0.0022(8)
O5	0.0000(8)	0.0056(8)	0.0016(8)
O6	0.0004(8)	0.0021(8)	0.0007(8)
O7	–0.0026(8)	–0.0003(8)	0.0014(8)
O8	0.0007(8)	0.0002(8)	0.0024(8)
O9	–0.0005(8)	0.0014(8)	0.0016(9)
O10	0.0010(8)	0.0002(8)	0.0013(8)
O11	–0.0010(8)	0.0008(8)	–0.0009(8)
O12	0.0040(9)	0.0000(8)	–0.0007(9)
O13	–0.0006(9)	0.0038(8)	0.0031(9)
O14	–0.0016(9)	0.0017(8)	0.0007(9)

**Table 4:** Cerium-oxygen and boron-oxygen distances /pm in Ce_4_B_14_O_27_, calculated with the single-crystal lattice parameters

Ce1–O13	238.4(2)	Ce2–O14	231.9(2)	B1–O1	145.4(3)
Ce1–O5	244.5(2)	Ce2–O9	237.8(2)	B1–O2	147.9(4)
Ce1–O8	255.0(2)	Ce2–O13	248.6(2)	B1–O3	148.5(4)
Ce1–O4	257.4(2)	Ce2–O10	251.7(2)	B1–O4	149.4(4)
Ce1–O3	261.7(2)	Ce2–O11	253.2(2)	Ø =	147.8
Ce1–O2	261.8(2)	Ce2–O14	253.2(2)		
Ce1–O6	267.5(2)	Ce2–O2	260.3(2)	B2–O6	145.0(4)
Ce1–O4	275.8(2)	Ce2–O12	284.1(2)	B2–O5	145.4(4)
Ce1–O1	283.1(2)	Ø =	252.6	B2–O7	147.4(4)
Ce1–O7	285.9(2)			B2–O4	148.8(4)
Ø =	263.1			Ø =	146.7
					
B3–O5	144.3(4)	B4–O9	144.5(4)	B5–O14	133.9(4)
B3–O9	146.0(4)	B4–O11	146.1(4)	B5–O7	137.3(4)
B3–O8	148.4(4)	B4–O10	148.6(4)	B5–O10	141.4(4)
B3–O2	148.8(4)	B4–O12	149.3(4)	Ø =	137.5
Ø =	146.9	Ø =	147.1		
					
B6–O13	134.7(4)	B7–O6	136.5(4)		
B6–O11	139.4(4)	B7–O3	136.7(4)		
B6–O12	139.5(4)	B7–O8	136.7(4)		
Ø =	137.9	Ø =	136.6		

**Table 5:** Selected interatomic angles /° in Ce_4_B_14_O_27_, calculated with the single-crystal lattice parameters

O1–B1–O2	105.5(2)	O6–B2–O5	103.0(3)
O1–B1–O3	116.4(3)	O6–B2–O7	113.4(3)
O2–B1–O3	108.2(3)	O5–B2–O7	114.3(3)
O1–B1–O4	108.9(2)	O6–B2–O4	112.8(3)
O2–B1–O4	113.8(2)	O5–B2–O4	109.7(2)
O3–B1–O4	104.3(2)	O7–B2–O2	104.0(3)
Ø =	109.5	Ø =	109.5
			
O5–B3–O9	112.9(3)	O9–B4–O11	112.7(3)
O5–B3–O8	110.9(3)	O9–B4–O10	112.1(2)
O9–B3–O8	112.6(3)	O11–B4–O10	103.8(3)
O5–B3–O2	110.5(3)	O9–B4–O12	107.3(3)
O9–B3–O2	103.5(3)	O11–B4–O12	112.1(2)
O8–B3–O2	106.0(2)	O10–B4–O12	108.9(3)
Ø =	109.4	Ø =	109.5
			
O14–B5–O7	117.1(3)	O13–B6–O11	122.5(3)
O14–B5–O10	121.8(3)	O13–B6–O12	116.2(3)
O7–B5–O10	121.1(3)	O11–B6–O12	121.2(3)
Ø =	120.0	Ø =	120.0
			
O6–B7–O3	117.8(3)		
O6–B7–O8	120.3(3)		
O3–B7–O8	121.8(3)		
Ø =	120.0		

Further details of the crystal structure investigations may be obtained from the Fachinformationszentrum Karlsruhe, 76344 Eggenstein-Leopoldshafen, Germany (Fax: +49-7247-808-666; E-Mail: crysdata@fiz-karlsruhe.de, http://www.fiz-karlsruhe.de/request for deposited data.html) on quoting the depository number CSD-425017 http://www.ccdc.cam.ac.uk/data_request/cif.

## Results and Discussion

### Crystal Structure of Ce_4_B_14_O_27_

**Figure 2:**
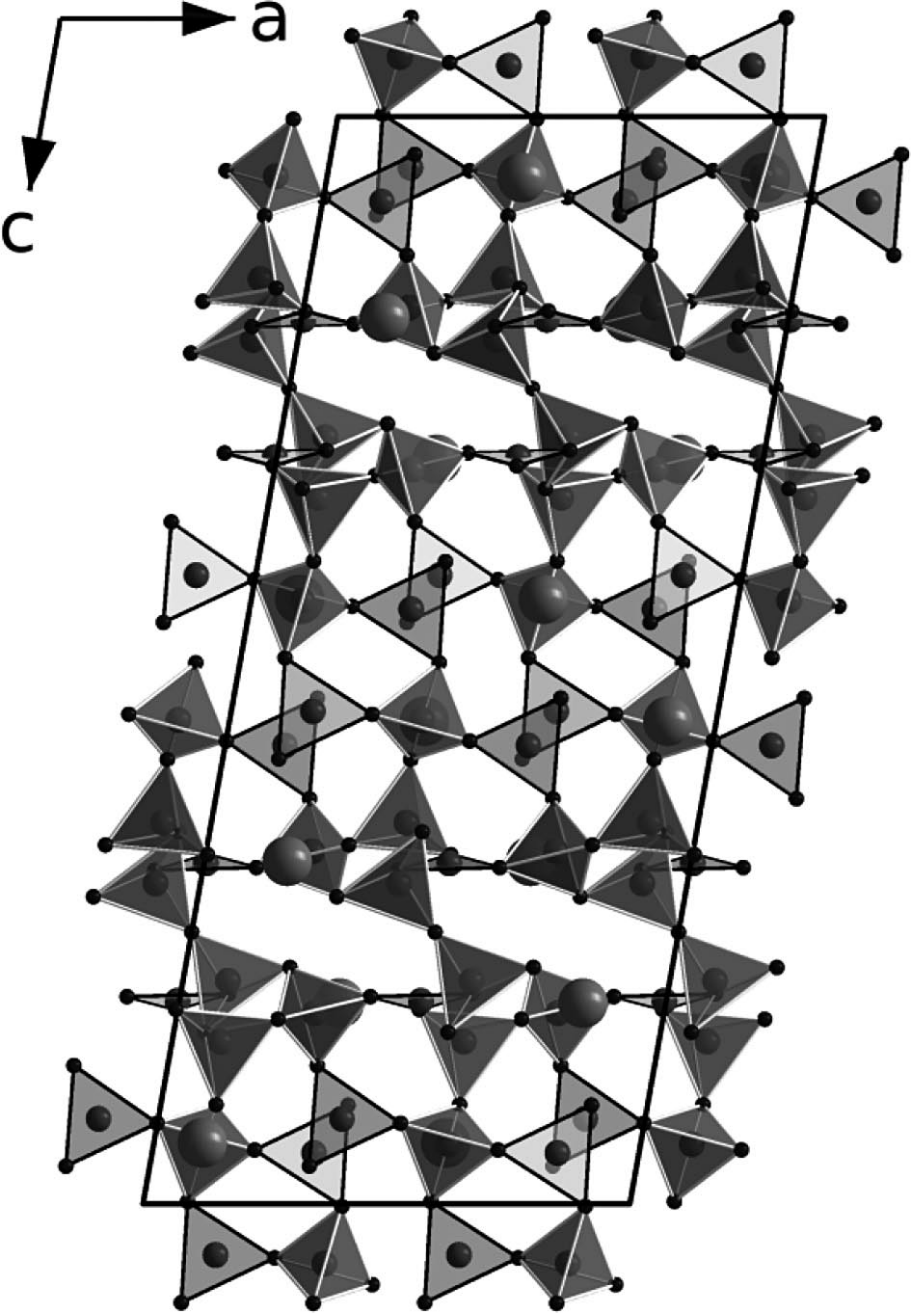
Crystal structure of Ce_4_B_14_O_27_ along [0$\bar{1} 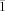 0].

**Figure 3:**
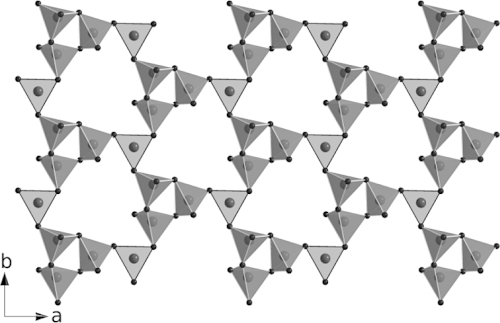
A layer built up of so called “dreier” rings and trigonal [B(7)O_3_]^3–^ groups.

**Figure 4:**
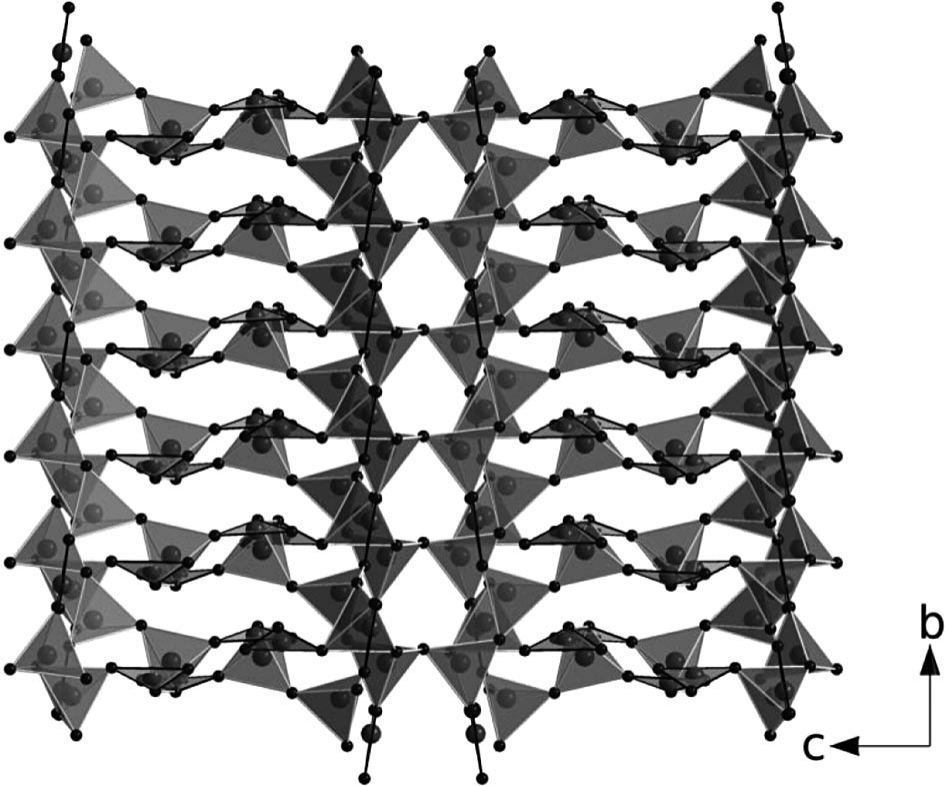
Two layers which are condensed to a double layer.

**Figure 5:**
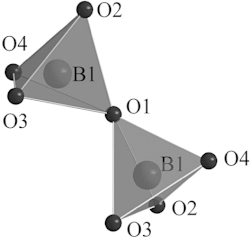
Linking [B_2_O_7_]^8–^ group.

**Figure 6:**
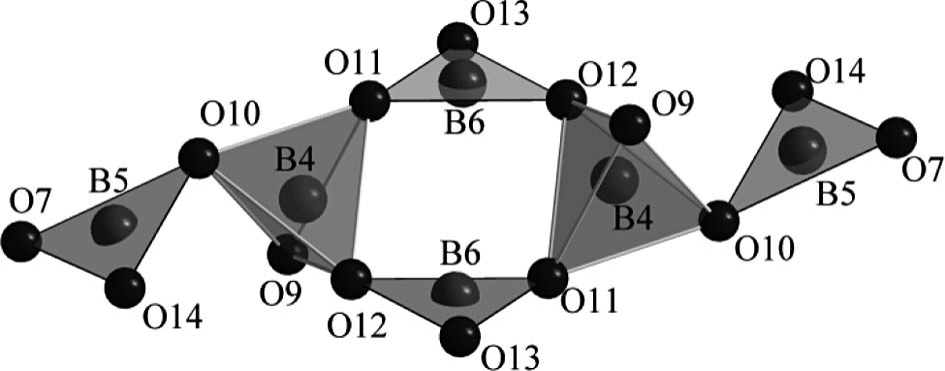
Condensed [B(4)O_4_]^5–^ and [B(5, 6)O_3_]^3–^ groups, which link the double layers.

**Figure 7:**
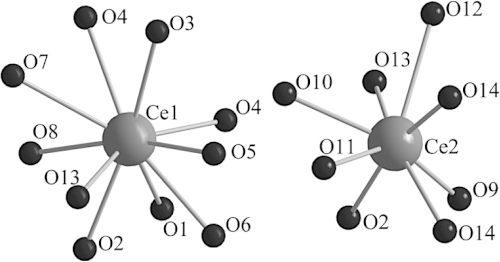
Coordination spheres of the Ce^3+^ ions in Ce_4_B_14_O_27_.

The structure of Ce_4_B_14_O_27_ consists of a highly condensed boron-oxygen network and trivalent cerium ions. Figure 2 shows the structure along [0 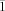 0]. The boron-oxygen network is composed of linked trigonal [BO_3_]^3–^ and tetrahedral [BO_4_]^5–^ groups. Four of the seven crystallographically different boron atoms are coordinated by four oxygen ions [B1–B4; *d*(B–O) = 144.3(4)–149.4(4) pm, ∠O–B–O 133.9(4)–141.4(4)°], while the remaining boron atoms build up trigonal groups [B5–B7; *d*(B–O) = 133.9(4)–141.4(4) pm, ∠O–B–O 116.2(3)–122.5(3)°]. All cerium–oxygen distances and boron–oxygen distances are listed in Table 4. The mean values of the boron-oxygen distances (146.7–147.8 pm for tetrahedral coordinated boron atoms and 136.6–137.9 pm for trigonal-planar boron atoms) correspond well with the known average values for B–O distances in [BO_4_]^5–^(147.6 pm) and [BO_3_]^3–^ (137.0 pm) groups.^29^–^31^ The oxygen–boron–oxygen angles of the trigonal [BO_3_]^3–^ and tetrahedral [BO_4_]^5–^ groups are listed in Table 5 and correspond well to the expected angles of tetrahedral- and trigonal groups. Three tetrahedral [BO_4_]^5–^ groups (B1–B3) are condensed via shared corners to so called “dreier” rings.^32^. These [B_3_O_9_]^9–^ groups are linked via trigonal [BO_3_]^3–^ groups (B7) to layers in the *ab* plane as shown in Figure 3. Two of these layers condense via the O(1) atom to a double layer (Figure 4). The resultant double tetrahedron [(B1)_2_O_7_]^8–^ is shown in Figure 5. These double layers are linked via strands of condensed [B(4)O_4_]^5–^ and [B(5, 6)O_3_]^3–^ groups to a three-dimensional network (Figure 6). The crystal structure of Ce_4_B_14_O_27_ contains two crystallographically distinguishable rare-earth ions. The rare-earth ion Ce1 is surrounded by ten oxygen atoms between 238.4(2) and 285.9(2) pm with a mean value of 263.1 pm, whereas Ce2 is coordinated by eight oxygen atoms between 231.9(2) and 284.1(2) pm with a mean value of 252.6 pm. Figure 7 displays the coordination spheres of the cerium ions. For a more detailed description of the structure, the reader is referred to the description of the isotypic compound La_4_B_14_O_27_.^16^ In this paper, we briefly compare Ce_4_B_14_O_27_ to the isotypic phase La_4_B_14_O_27_.

The MAPLE values (*MA*delung *P*art of *L*attice *E*nergy)^33^–^35^ were calculated from the crystal structure to compare them with the MAPLE values received from the summation of the binary components A-type Ce_2_O_3_^36^ and the high-pressure modification B_2_O_3_-II.^37^ The value of 182961 kJ**·**mol^–1^ was obtained in comparison to 181866 kJ**·**mol^–1^ (deviation = 0.6 %), starting from the binary oxides [2 × Ce_2_O_3_ (14150 kJ**·**mol^–1^) + 7 × B_2_O_3_-II (21938 kJ**·**mol^–1^)].

Furthermore, the bond-valence sums of Ce_4_B_14_O_27_ were calculated from the crystal structure for the rare-earth ions, using the bond length/bond-strength concept (*Σ*V).^38^,^39^ The calculation revealed values of: +2.95 (Ce1) and +3.12 (Ce2), which fit well for the formal ionic charges. For the boron ions, the values vary between +2.91 and +3.09. The oxygen ions show values of –1.90 to –2.15.

The comparison of the lattice parameters *a*, *b*, *c*, and *β* [*a* = 1117.8(2), *b* = 640.9(2), *c* = 2531.7(5), *β* = 100.2(1)° for Ce_4_B_14_O_27_ and *a* = 1120.84(9), *b* = 641.98(6), *c* = 2537.2(2), *β* = 100.125(8)° for La_4_B_14_O_27_] reveals the typical rise from cerium compounds to lanthanum compounds corresponding to the slightly larger size of La^3+^ compared to Ce^3+^. No greater deviations of the bond lengths and angles are observed. The coordination numbers of the rare-earth ions are equivalent.

### Physical Properties of Ce_4_B_14_O_27_

#### Raman Spectroscopy

Confocal Raman spectra of single crystals of Ce_4_B_14_O_27_ were measured in the range of 100–6000 cm^–1^ with a HORIBA LABRAM HR-800 Raman micro-spectrometer under a 100 × objective (numerical aperture N.A. 0.9, Olympus, Hamburg, Germany). The crystal was excited by the 532.22 nm emission line of a 30 mW Nd:YAG laser (green). The laser focus on the sample surface was ca. 1 μm. The scattered light was dispersed by a grating with 1800 lines per mm and collected by a 1024 × 256 open electrode CCD detector. Third order polynomial background subtraction, normalization, and band fitting by Gauss-Lorentz functions were done by the LABSPEC 5 software (HORIBA).

**Figure 8:**
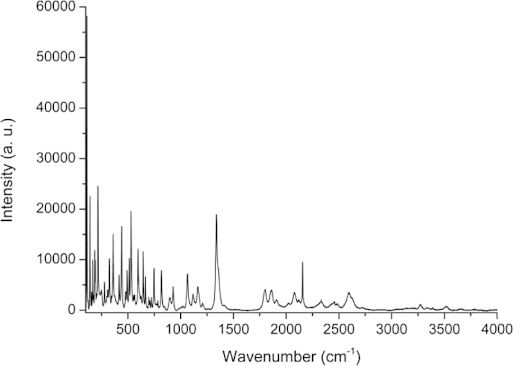
Raman spectrum of Ce_4_B_14_O_27_ in the range of 100 to 4000 cm^–1^.

**Figure 9:**
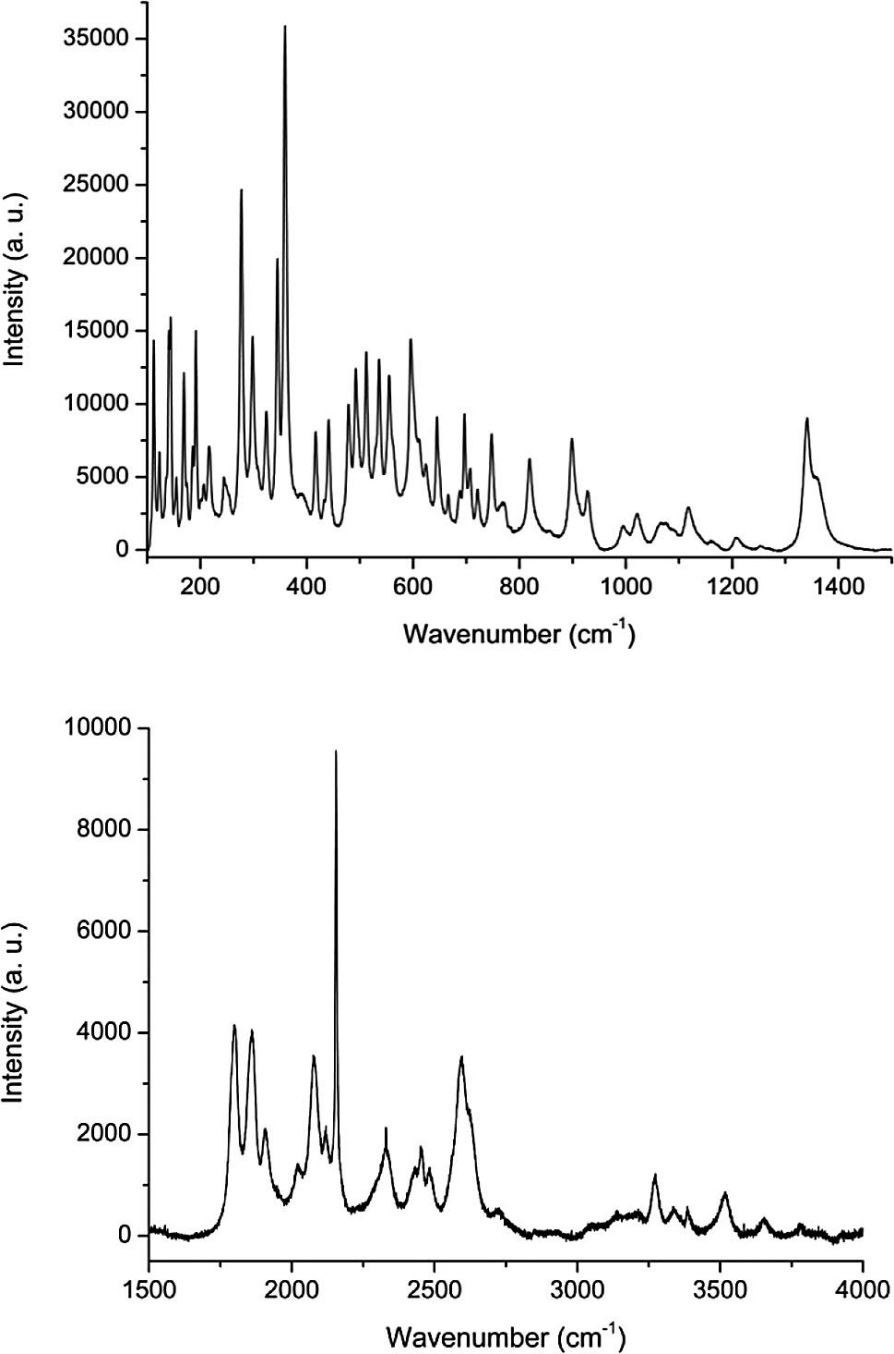
Raman spectrum of the compound Ce_4_B_14_O_27_ between 100 and 1500 cm^–1^ (top) and between 1500 and 4000 cm^–1^ (bottom).

Figure 8 shows the Raman spectrum of Ce_4_B_14_O_27_ from 100 to 4000 cm^–1^. In the range of 3000 to 3600 cm^–1^, no OH or water bands could be detected. Bands around 900 cm^–1^ in oxoborates are usually assigned to stretching modes of the [BO_4_]^5–^ groups. However, the trigonal [BO_3_]^3–^ groups are expected at wavenumbers above 1100 cm^–1^.^5^,^40^-^43^ The range between 100 and 1500 cm^–1^ is displayed in Figure 9 (top) and the range between 1500 and 3000 cm^–1^ in the bottom of Figure 9. Bands at wavenumbers smaller than 500 cm^–1^ can be assigned to Ce–O bonds, to lower wavenumbers shifted bending and stretching modes of the tetrahedral [BO_4_]^5–^ groups, as well as lattice vibrations. As expected, bands between 800 and 1800 cm^–1^ are observed due to the presence of trigonal [BO_3_]^3–^ and tetrahedral [BO_4_]^5–^ groups, whereas vibrational modes above 1200 cm^–1^ generally refer to trigonal [BO_3_]^3–^ groups. The large variation of B–O distances inside the [BO_4_]^5–^ and [BO_3_]^3–^ groups leads to various modes and based on the deviation of the distances from the ideal B–O distance to a large shift of the bands.

## Conclusions

With the successful synthesis of Ce_4_B_14_O_27_, a new cerium polyborate and furthermore the first isotypic compound to La_4_B_14_O_27_ was synthesized and characterized. In accordance with the relatively low applied pressure of 2.6 GPa, the boron oxygen network is built up by trigonal [BO_3_]^3–^ and tetrahedral [BO_4_]^5–^ groups. Four of the seven crystallographically different boron atoms are coordinated by four oxygen ions. The application of similar synthetic conditions to heavier rare-earth elements could lead to additional isotypic compounds and will be studied in the future.
